# Pathways to ischemic neuronal cell death: are sex differences relevant?

**DOI:** 10.1186/1479-5876-6-33

**Published:** 2008-06-23

**Authors:** Jesse T Lang, Louise D McCullough

**Affiliations:** 1 Departments of Neurology and Neuroscience, The University of Connecticut Health Center, Farmington, CT, USA; 2 The Stroke Center, Hartford Hospital, Hartford, CT, USA

## Abstract

We have known for some time that the epidemiology of human stroke is sexually dimorphic until late in life, well beyond the years of reproductive senescence and menopause. Now, a new concept is emerging: the *mechanisms *and *outcome *of cerebral ischemic injury are influenced strongly by biological sex as well as the availability of sex steroids to the brain. The principal mammalian estrogen (17 β estradiol or E2) is neuroprotective in many types of brain injury and has been the major focus of investigation over the past several decades. However, it is becoming increasingly clear that although hormones are a major contributor to sex-specific outcomes, they do not fully account for sex-specific responses to cerebral ischemia. The purpose of this review is to highlight recent studies in cell culture and animal models that suggest that genetic sex determines experimental stroke outcome and that divergent cell death pathways are activated after an ischemic insult. These sex differences need to be identified if we are to develop efficacious neuroprotective agents for use in stroke patients.

## Background

Stroke affects 15 million people worldwide each year, and is the leading cause of disability in the United States. The epidemiology of ischemic stroke is sexually dimorphic in that ischemic events occur with greater frequency in men vs. women regardless of country-of-origin and ethnic culture [1]. The underlying mechanisms involved in these sex differences remain unclear [2] but exposure to gonadal hormones, particularly estrogen, has been thought to play a major role [3,4]. In experimental stroke studies, female animals suffer less damage from an induced stroke than males, an effect that can be reversed in part by ovariectomy [5]. However, despite preclinical and observational evidence of a protective role for estrogen, recent randomized clinical trials such as the Women's Health Initiative (WHI) have failed to translate the beneficial effects of estrogen into a viable therapy for stroke prevention in post-menopausal women, as treatment with estrogen led to an unexpected increase in stroke rates [6]. In addition, women continue to have a decreased incidence of stroke compared to men well beyond (>20 years) the menopause, suggesting that not all the observed "female protection" is mediated by steroids.

It is becoming clear that innate differences in stroke risk exist between the sexes that are *independent of hormone exposure *[7]. Hormone-independent sexual dimorphism has been described in pediatric stroke studies as well as in experimental animal models of neonatal hypoxic-ischemic encephalopathy (HIE) [8]. For example, male sex is a significant risk factor in childhood stroke and is linked to higher mortality after ischemic stroke in boys relative to girls [9]. Female pre-term neonates have better survival and fewer handicaps compared to males [10], and animal studies show clear sex differences in outcome in models in which hormone levels are similar between the sexes [11]. Much less evidence is available for adult animals, as the confounding effect of hormones has made this a difficult area of investigation [4,12] and it remains relatively understudied. There is abundant preclinical evidence that estrogen protects ischemic brain [13-15], however very little work has examined male hormones as a possible mediator of the innate male sensitivity to cerebral ischemia. One recent study has shown that removal of androgens protects the male brain from damage induced by middle cerebral artery occlusion (MCAO), which is reversed by testosterone replacement [16], suggesting that testosterone exposure could be deleterious. It is important to note that the effects of hormones can never be completely eliminated, even in neonatal models of ischemia. It has been well described that some sex-related traits may be influenced by variations in hormonal exposure during fetal development due to intrauterine positioning [17]. Ischemic sensitivity could be related to prenatal hormonal exposure (ie. testosterone, progesterone or estrogen) even in *in vitro *studies that utilize neuronal cultures derived from embryonic cells or neonatal slice studies.

Modeling ischemic "stroke" in the laboratory setting is also difficult. Results from cell culture systems after ischemic or excitotoxic insults and animal studies of induced stroke should be interpreted with some caution. Stroke incidence and functional outcome measures commonly used in our stroke patients are clearly distinct from the pre-clinical measurements of "infarct size", short term histological assessment, and simple behavioral endpoints frequently used in rodent studies. Additionally, although it is becoming accepted that sex differences are present in acute stroke outcomes in both animals and humans, possible sex differences in repair and regeneration after injury have yet to be addressed.

Due to cost constraints and high mortality, stroke researchers also often utilize only young animals (8–12 weeks for rodents) with induced stroke examined at 24 or 72 hours after injury. This clearly does not adequately reflect the clinical disease, as the vast majority of spontaneously occurring strokes occur in older individuals with multiple risk factors (i.e., hypertension, diabetes etc.) who accrue long-term disability. These issues are becoming increasingly recognized as major blocks to translational research [18,19]. Only one study has examined sex differences in naturally occurring stroke in rodents. Yamori et al examined spontaneous stroke incidence in over 2000 genetically hypertensive and stroke-prone animals and found results consistent with the intrinsic female protection seen in other models. Female rats had longer life expectancies compared to age-matched males and low rates of cerebral hemorrhage and vascular lesions until an advanced age [20]. Some of these effects can be attributed to estrogen, but other possible mediators of these sex differences need to be evaluated.

So what is the etiology of these sex differences? Over the past five years, data has emerged from *in vivo *and *in vitro *studies that demonstrate that ischemic cell death pathways are fundamentally different in the male and female brain. It appears that females are exquisitely sensitive to caspase-mediated cell death, whereas cell death in males is triggered by caspase-independent pathways involving apoptosis-inducing factor (AIF) and Poly(ADP-ribose) polymerase (PARP) activation [4,21,22]. So why is it important to further investigate these sex differences? Even if we discover that differences exist, does this have any relevance to clinicians developing drug therapies or treating patients? The answer becomes clear when the few experimental neuroprotective studies that have used both sexes in their work are reviewed. Several clinically relevant neuroprotective agents that are in development for the treatment of stroke and HIE have shown clear sexual dimorphic responses i.e., PARP inhibitors[4], erythropoietin [23] hypothermia [24] and caspase inhibitors [22]. In fact, in adult mice, agents designed to interfere with PARP signaling actually worsened outcome in females, although these agents had a dramatic protective effect in treated males. These effects were independent of estrogen exposure as they were seen in ovariectomized as well as intact females [4]. These studies are extremely relevant to the treatment of adult stroke patients with neuroprotective agents, as the vast majority of women experiencing an ischemic event are post-menopausal, with low circulating estrogen levels [4].

An important new concept emerging in this field is that therapies for stroke operate in a different genetic background in women and men [3]. This review aims to summarize the recent literature on sex differences in animal models of ischemic stroke as well as in ischemic cell death in culture systems, with a focus on *hormone-independent mechanisms*. Sex differences have also been reported in other organ systems, the best studied being the kidney and heart; however, most of the work has investigated direct hormonal protection rather than intrinsic sex differences [25-27]. This is not to suggest that the brain is the only area where sex differences play an important role in outcome. In one recent study sex differences were described in neonatally-derived cardiomyocytes, demonstrating the wide relevance of these sex differences to other organ systems [28]. There is also considerable clinical literature suggesting sex differences exist in clinical stroke epidemiology, prevention and treatment. Many of these effects are likely related to hormone exposure, and are summarized in an excellent recent review on this topic, [29] yet discerning which effects are attributable to hormones and which are intrinsic to gender is essential. Advancing our knowledge of the mechanisms of ischemic cell death and neuroprotective therapies is an important goal in both sexes in order to optimize treatments for stroke. Many researchers are unaware of the potential confounding effects of sex differences. Much of the preclinical work in stroke continues to focus on young male animals and mixed sex cell culture systems despite the Stroke Therapy Academy Industry Roundtable (STAIR) recommendations that neuroprotective studies be performed in both male and female animals [30]. It is imperative that investigators are aware of the potential for erroneous conclusions when attempting to translate promising experimental findings into a clinical population at risk for stroke which includes women.

### Programmed cell death

Currently, the only FDA-approved treatment for stroke is administration of tissue plasminogen activator (tPA), which degrades the fibrin clot blocking blood flow to the brain tissue [31]. Unfortunately this treatment is only approved for the 1^st ^three hours after onset of stroke. Due to this short time window, researchers and clinicians are focusing on treatments that can be administered several hours after ischemic onset, specifically targeting a slower cell death pathway than necrosis: apoptosis. Apoptosis plays a key role in stroke-related cell death, yet no drugs targeting this pathway have been approved for clinical use. New experimental data points to significant sex-based differences in the activation and execution of apoptosis between male and female animals in response to stroke. The ability to discriminate such differences may help increase success of these drugs in clinical trials.

**Figure 1:**
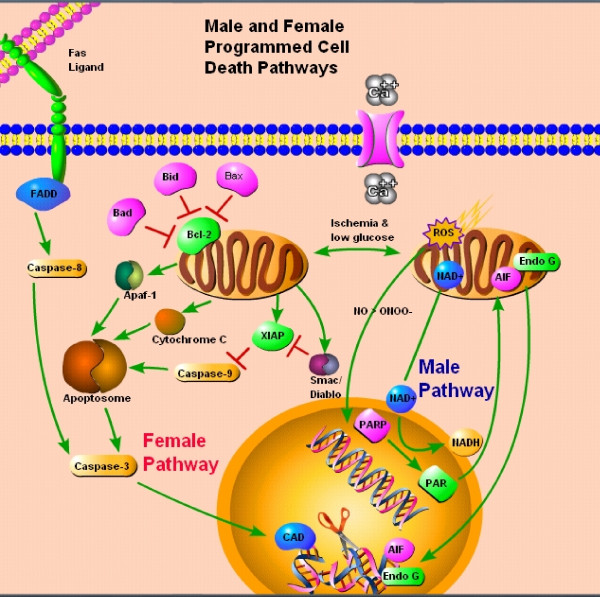
Proposed model of cell death pathways in response to ischemia in females and males.

Several drugs inhibit apoptosis, a normal process used during development that occurs to a smaller degree as we age [32]. Quantifying the degree of apoptotic cell death is difficult, as subtle changes in the techniques or time points used may affect the results and initiating events often occur concurrently [33]. Specifically, apoptosis can be triggered through a number of factors either through an "intrinsic" mitochondrial mediated or "extrinsic" cell death receptor pathway [34]. In stroke, the intrinsic pathway is usually initiated by the release of cytochrome C from the mitochondria (Figure 1). This is followed by the formation of the apoptosome, caspase cleavage with subsequent amplification of downstream targets, and the eventual cleavage of DNA and structural molecules leading to the death of the cell [33]. Alternatively, DNA damage may trigger over-activation of poly-ADP-ribose polymerase (PARP), with corresponding release of AIF and endonuclease G from the mitochondria eventually terminating in cell death [35]. The relationship between these two forms of apoptosis is still under examination, yet substantial research exists demonstrating that these pathways can be activated in a sexually dimorphic way. More importantly, agents that interfere with activation of a specific triggering event in each pathway (ie., PARP vs. the caspase cascade) have very different results in male or female animals or tissue, suggesting that intrinsic differences exist based solely on sex.

Many of the early observed clinical and experimental sex differences were explained by the pronounced effect that gonadal hormones confer in stroke. Numerous studies have documented the protective role of estrogen in stroke and *in vitro *oxygen-glucose deprivation models [36-38]. Physiological levels of sex hormones may mask innate gender differences, but these differences may be uncovered when hormone levels are equalized between sexes. This necessitates ovariectomizing (OVX) adult females and thus explains the substantial emphasis placed on neonatal animal research in this area. The utilization of neonatal animals allows for the investigation of cell death independently from the effects mediated by hormones [39]. Here we will briefly discuss neonatal, adult, and *in vitro *studies that have formed the foundation of the hypothesis that cell death is sexually dimorphic, even at a molecular level. A summary of articles that specifically examined sex-related differences in response to ischemic stress in the brain is listed in table 1.

**Table 1:** Studies examining gender-differences independent of gonadal hormones in response to cellular stressors

**Author**	Age*	**Insult**	**Treatment/drug/mutation**	**Sex difference**	**Key molecules**
Du, L [21]	Neurons	Cytotoxic agents	estradiol, NMDA antagonist, z-VAD, PARP inhibitor, antioxidants	Yes	AIF, cytochrome C, glutathione
Hagberg, H [53]	Neonatal	50 minutes unilateral hypoxia-ischemia	PARP-1 -/-	Yes	NAD+, PAR
Heyer, A [37]	Neurons	15 hr. Hypoxia	testosterone	Yes	sex hormones, estrogen receptors
Kitano, H [60]	Neonatal and adult	2 hr. MCAO	isoflurane PC, Akt KO	Yes	Akt, NIPK
Li, H [48]	Neurons	Oxygen-glucose deprivation & NMDA	7-nitroindazole, estradiol	Yes	SOD, NO(x)
Li, K [57]	Juvenile	Embolic infarct	None	Yes	various cytokines
Liu, M [38]	Astrocytes	Oxygen-glucose deprivation & cytotoxic agents	Arimidex, estradiol	Yes	cyp19 mRNA, aromatase activity
Mabley, J. G. [12]	Adult	LPS injections	OVX, PARP-1 -/-	Yes	TNF, PAR, Erα
Mcullough, L. D. [4]	Adult	90 min. MCAO	nNOS -/-, PARP- 1-/-, OVX, PJ-34, 7-nitroindozole	Yes	eNOS, iNOS, nNOS
Nijboer, CH [11]	Neonatal (P7)	2 hr. Hypoxia-ischemia	2-iminobiotin	Yes	AIF, cytochrome C, caspase-3
Nijboer, CH [51]	Neonatal (P3)	2 hr. Hypoxia-ischemia	2-iminobiotin	Yes	AIF, cytochrome C, caspase-3, HSP70
Park, EM [59]	Adult	20 min. MCAO	OVX, PARP-1 -/-, aminoguanidine, iNOS-/-	Mixed	iNOS
Renolleau, S [22]	Neonatal	50 min. MCAO	Q-VD-Oph	Yes	cytochrome C, caspase-3, caspase-1
Wen, TC [23]	Neonatal	permanent MCAO	Erythropoietin	Yes	None
Zhang, L [42]	Neurons	None	None	Yes	Phospho-ERK1, Akt, Bcl-2
Zhu, C [54]	Neonatal	45 min. Unilateral hypoxia-ischemia	Q-VD-OPh, Edaravone, Harlequin mutation	No	AIF, cytochrome C, caspase-3, PAR
Zhu, C [56]	Neonatal and Adult	45–65 min. Unilateral hypoxia-ischemia	None	Mixed	AIF, cytochrome C, caspase-3, PAR, nitrotyrosine

*all studies were performed on rats or mice

### Sex differences in cell culture models

Early studies examining adult rodents found that OVX females displayed similar infarct volumes compared to age-matched males after 2 hr. MCAO [40]. Additionally, males supplemented with equine estrogens had smaller strokes than untreated males [41]. This supported the idea that estrogen was the principle cause of dichotomous stroke outcome between sexes. However, examining survival rates of embryonically-derived cortical neurons (DIV 14) separated by sex revealed that female neurons in both the cortical plate and the ventricular zone survived longer than male neurons [42]. Furthermore, phospho-ERK1 and Akt levels were higher in female neurons, suggesting that different pro-survival pathways could be activated in XX vs. XY cells independently of hormone exposure. The vast majority of previous studies specifically examining sexual dimorphism focused on sexual differentiation and the organizational effect of hormones on areas involved in reproductive and mating/maternal behaviors. It is becoming increasingly clear that sexual dimorphisms can be the result of the genetic complement of the cell and occur completely independently of hormonal exposure [43,44] in areas that are unrelated to sexual development. Indeed, sex-differences in gene expression occur prior to gonadal differentiation [45-47]. The possible consequences of genetic contributions (XX vs. XY) to ischemic sensitivity have only recently been investigated.

Sex differences in the response to oxygen-glucose deprivation (OGD) or ischemic-like insults have been evaluated *in vitro *(see Table 1). A consistent sex difference after either OGD or NMDA exposure has been seen in hippocampal slice cultures; slices derived from female post-natal (PND 7) pups were intrinsically protected compared to slices derived from male animals [48]. Primary rat female hippocampal neurons were also more resistant to hypoxia than male neurons [37]. Sex differences in sensitivity to ischemia have also been recently described in post natal astrocytes [38]. In a study using cytotoxic agents to induce cell death, female neurons demonstrated greater resistance to nitrosative stress than male neurons [21]. Additionally, male and female neurons responded differently to drugs targeting specific proteins and pathways including a PARP-1 inhibitor and z-VAD.fmk, a pan-caspase inhibitor. Higher levels of AIF, a major downstream mediator of PARP's cytotoxic effects, were observed in the nucleus of male neurons, while higher levels of cytosolic cytochrome C, an initiating event in the intrinsic caspase cascade, were observed in female neurons [21]. Similar results in *in vivo *stroke models (see below) support this concept: male cell death after stroke is mediated in large part by the activation of neuronal nitric oxide synthase (nNOS) with subsequent activation of PARP, whereas female cell death is triggered by cytochrome C and caspase activation. If this is true, then drugs that interfere with nNOS/PARP activation are unlikely to benefit female-derived neurons, and conversely agents that interfere with caspase activation are unlikely to benefit male-derived neurons. Evidence for this is accumulating in the literature both *in vitro *and *in vivo*. Treatment of hippocampal slices with a nNOS inhibitor had no effect in female slices (PND 7; DIV 13) after OGD [48], but protected male neurons. Similar sex dichotomies have been observed in splenocytes, suggesting that this may be a ubiquitous sex-difference in response to stress in cells outside of the central nervous system as well [21]. These sex differences may be an important but relatively ignored source of variation in mixed sex cell cultures. Single sex cultures are much more time consuming, but this recent data does highlight potential translational problems when only mixed sex neuronal or astrocytic cultures are examined. These *in vitro *studies set the stage for subsequent *in vivo *examinations of sex differences after stroke. From this work it is becoming apparent that there is a possible "switch-point" for cell death leading to a cascade of death events that differ in males and females (see Figure 1).

### Sex differences in neonates

There is considerably more data on sex-divergent cell death in neonatal stroke models than what currently exists in the adult animal literature. The clinical phenomenon of female protection after neonatal injury has been well described, and is much less likely to be due to hormonal differences. This has prompted investigators to examine sex differences earlier than we have in adult models. Most sex differences in adults have been ascribed to estrogen; this issue has been largely avoided by researchers who often utilize only males in experimental studies to avoid the variability in ischemic outcome seen in cycling females [13,49]. One way to address these issues is to examine models in which hormonal exposure is minimal; in neonatal animals. A series of recent studies has evaluated the protective effects of 2-iminobiotin (2-IB) in a neonatal HI model [50]. This agent was protective and reduced damage 6 weeks after injury in post-natal day 12 rats, an effect attributed to 2-IB's actions as a putative neuronal and inducible nitric oxide synthase (nNOS and iNOS) inhibitor. However, treatment with 2-IB did not lead to decreased levels of nitrotyrosine, a marker of activation of NO. In contrast, 2-IB prevented hypoxia-induced increases in cytochrome C levels. If the effects of 2-IB were on caspase pathways, then it would be expected that female animals would benefit more than males. The original experiments were not designed to look at sex effects, but these authors subsequently examined their data by sex and discovered that female pups benefitted from treatment, whereas no effect was seen in males [50]. Later studies further confirmed these findings [51]. Female post-natal day 7 rats had reduced long-term brain damage whereas no treatment effect was seen in males [51]. Furthermore, elevated levels of AIF were only observed in males, and these levels were unaffected by 2-IB treatment. Alternatively, only females displayed decreased levels of cytochrome C and cleaved caspase-3 in response to 2-IB treatment. Similar results were reported after repeating the experiment on P3 rats, with only females displaying protection with 2-IB treatment [11]. This study, however, found no difference in cytochrome C release or HSP70 between sexes.

The question remained as to whether these differences are secondary to enhanced caspase activation in females, or an intrinsic female sensitivity to caspase-induced cell death. Recently, it was shown that P7 female rats were dramatically protected when given the pan-caspase inhibitor Q-VD-OPh at reperfusion after a 50 min. focal injury, while males showed no protection from the treatment [22]. Males had a large increase in cytolosic cytochrome C levels (implying its release from the mitochondria) between 6–12 hrs. after reperfusion, whereas females had a gradual appearance of cytosolic cytochrome C which peaked at 16 hrs. Furthermore, females had significantly higher levels of cleaved caspase-3 than males, which peaked at 12 hrs. after reperfusion. Sex differences in mitochondrial membrane permeability and caspase pathway activation could explain these findings but the underlying mechanisms leading to these sex differences remains unknown. Sex differences have also been seen in behavioral outcome and infarct size after administration of the putative neuroprotective agent, erythropoietin (EPO). A permanent middle cerebral artery occlusion (MCAO) was performed in male and female neonatal rats given EPO or vehicle and infarct volume at 6 weeks and functional recovery at 12 weeks were examined. A greater reduction in infarct volume as well as improved functional recovery was observed in females compared to males [23]. Although specific cell death molecules were not measured, a clear sex difference in response to this potential neuroprotective agent that is already utilized in clinical populations emphasizes the clinical importance of these investigations.

These studies have begun to explore differences in caspase-mediated apoptosis between the sexes, yet the question of how caspase-independent cell death via PARP activation and AIF translocation differs by sex is also an important question. Although there is a considerable amount known about PARP and its role in post-ischemic brain injury [52], most of this data has been generated exclusively in male animals and cell cultures derived from both male and female embryos. A study in 2004 using neonatal PARP-1 deficient mice observed neuroprotection in males but not females in response to a unilateral hypoxia-ischemic injury [53]. Many of the deleterious effects of PARP activation are thought to be secondary to PARP-induced translocation of the pro-apoptotic molecule AIF from the mitochondria to the nucleus. Therefore, if sex differences are present in the response to PARP deletion, they may be secondary to changes in AIF. This does not appear to be the case in neonatal models. A recent study using P9 Harlequin (Hq) mutant mice, which have a 60% reduction in AIF expression, [54] demonstrated neuroprotection in both male and female Hq pups after HI insults. Neither male nor female Hq mutant mice had decreased caspase-3 activation or cytochrome C release after injury, suggesting that the NO/PARP/AIF pathway is distinct from the cytochrome C/caspase pathway. Additional experiments demonstrated that treatment with Q-VD-OPh in Hq mutant mice led to greater neuroprotection than the Hq mutation alone. In this study, the caspase inhibitor was given to both wild type (WT) and Hq mice of both sexes and appeared to lead to neuroprotection in both, but results were not dichotomized or analyzed by gender. Renolleau has demonstrated that caspase inhibition with Q-VD-OPh is ineffective in reducing injury in male neonatal WT mice [22]. These disparate findings could be due to differences in the ages, models, or doses used or simply be due to the fact that the study was not designed to directly evaluate sex differences. It is also possible that blocking the NO/PARP/AIF in the Hq mice unmasks a sensitivity to caspase-induced cell death in the male brain that is not usually present that can subsequently be inhibited by Q-VD-OPh. In adult models, the hq mutation decreases infarct in adult males [55] and has little effect in females (McCullough, et al unpublished results). Levels of AIF drop in the Hq mice as the animal matures (60% loss at P7 to 80% at 3 months), which could account for these differences.

Another study from the same laboratory examined both neonatal and intact adult mice and reported mixed results regarding sex differences [56]. Although they subjected the mice to various durations of unilateral HI, sex differences were only observed at specific durations and at certain ages. P9 males did have more AIF+ cells and more AIF-PAR co-localization in the nuclei of the striatum and cortex than females after HI. Females had higher cleaved caspase-3 levels than males after injury, yet the difference was only observed at 24 hours post-HI. Lastly, no sex difference in the amount of cytochrome C release at any time point was observed. These results suggest that the gender differences may be related to the duration of the ischemic insult or the model used. The higher amount of AIF release and cleaved caspase-3 levels in males and females respectively, does reinforce results from earlier studies. The disparity between elevated levels of cleaved caspase-3 in females with no sex difference in cytochrome C release may again indicate that other cytosolic molecules are present, possibly attenuating caspase activation in male cells.

### Sex differences in cell death in adult models

Acknowledging that rates of apoptosis differ among developmental ages, examining sex differences in adult models is critical, especially as the vast majority of stroke patients are older adults. One of the first studies to examine sex differences in experimental stroke outcome utilized an embolic infarct model and demonstrated smaller infarct volumes in female rats compared with males [57]. Females had an increased inflammatory response even after adjusting for infarct size. Caspases have been demonstrated to be activated after inflammatory insults [58], yet specific proteins were not measured in the previously described study [58].

The literature in the adult brain is sparser than the neonatal studies, and there has only been one paper on sex differences in NO/PARP in the ischemic brain to date [4] and one on sex differences in iNOS [59]. Neuroprotective agents do have differential effects based on sex, similar to what is seen in neonates. For example, isoflurane preconditioning decreased ischemic damage in male mice after MCAO but markedly increased infarction in female mice [60]. The mechanism for this remains unclear, but may involve activation of nNOS and PARP. PARP may be the key "switch point" in determining the mode of cell death. There is some evidence that sex differences also occur in adults in models other than stroke that are mediated by PARP activation. One interesting study examined the systemic inflammatory response to lipopolysaccharides (LPS) in males and females [12]. Female mice had a much less marked inflammatory response to systemic inflammation induced by endotoxin compared to males. Female mice were also resistant to endotoxin-induced mortality, an effect mediated in large part by estrogen, as this survival benefit was lost with ovariectomy. Deletion or inhibition of PARP-1 [12] decreased the inflammatory response in male animals but had no effect in female animals. The female's responsiveness to PARP inhibition was regained after ovariectomy, suggesting that female sex hormones may be acting in part by similar mechanisms as PARP, as loss of either ameliorated the sex difference. It was suggested that PARP may interact with the estrogen receptor (ER) to form a complex that binds to DNA, preventing the recognition of single strand breaks, (the main initiating event in PARP activation) and reducing PARP activation. Whether this putative ER binding plays a role in the ischemic brain is not yet known but data suggests that PARP deletion leads to loss of estrogen's neuroprotective effects after stroke.

**Figure 2:**
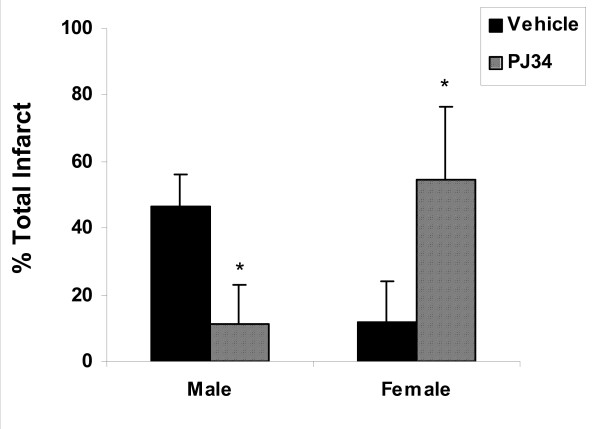
**The effects of the selective PARP-1 inhibitor PJ-34 in WT mice of both genders**. Treatment with PJ-34 at ischemic onset reduced total infarction in male mice compared to saline treated controls (*; p < .001). A significant increase in ischemic damage was seen in PJ-34 treated females compared to control (*; p < .001) [4].

**Figure 3:**
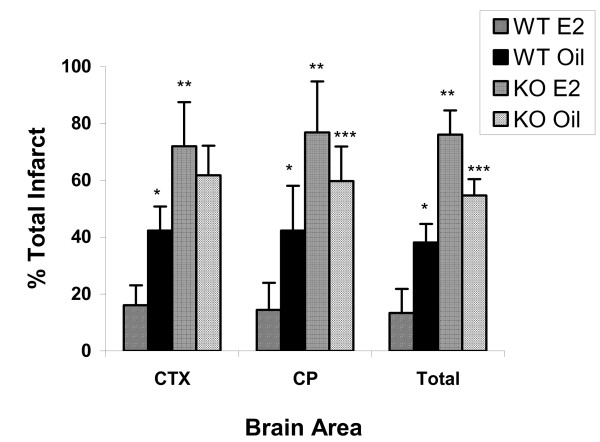
**Effect of estrogen on infarction volume in PARP-/- females**. Physiological levels of E2 were restored to ovariectomized (OVX) PARP-/- and WT female mice. WT females had significant reductions in total, cortical (CTX) and striatal (CP) infarct volumes after E2 replacement compared to oil treated WT females (*; p < .01). PARP-/- females demonstrated increased damage compared to WT (**; p < .01). Interestingly the neuroprotective effect of E2 was completely absent in PARP -/- females. E2 treatment exacerbated stroke damage; both striatal and total infarct volumes were significantly higher in E2 treated vs. oil treated PARP-/- mice (***; p < .05) [4].

The effects of nNOS and PARP-1 deletion or pharmacological inhibition in male and females after focal stroke has been evaluated [4]. Both the loss of PARP and nNOS or their inhibition protected males but not females. Pharmacological inhibition of PARP-1 surprisingly enhanced injury in ovary-intact females (Figure 2). Additionally, restoring estrogen to PARP-deficient OVX females exacerbated infarct volumes even further than PARP gene deletion alone (Figure 3). These results suggest that in the setting of PARP deficiency, estrogen may have deleterious effects. This data also implies that PARP-1 and NOS are endogenous neuroprotective pathways in the adult female brain. Perhaps the loss of PARP leads to enhanced "flow" through the caspase/cytochrome C pathway, to which females may be exquisitely sensitive? These hypotheses are currently being investigated by several laboratories. The interaction between pro-death cascades, sex, and hormones is a complex and intriguing line of inquiry that will have practical applications for clinicians involved in treating stroke patients. The findings from research examining gender differences in adult animals is less clear than research from neonates and culture studies, primarily due to the confounding influence that sex hormones exert. Additional studies focusing on adults and even more importantly, senescent animals will be needed to fully understand the implications for clinical therapies.

## Conclusion

Reviewing the literature on sex differences in response to stroke suggests that there is a dichotomous response between male and female animals that is independent of sex hormones. Although the exact nature of these differences has yet to be fully explained, it appears that the ability to maintain normal mitochondrial function, as well as the response to free radicals such as nitric oxide may play a critical role. Several studies have also shown sex differences in the activation of caspase-3, and recently the timing of cytochrome C release between the sexes. PARP-1 may provide a protective role in females while stimulating the production of PAR polymers and release of AIF from the mitochondria in males, leading to cell death. Sex differences are also clearly present in the efficacy of neuroprotective agents in our pre-clinical stroke models, and should be considered in clinical trial design. Lastly, a greater emphasis on studying older mice is necessary before clear conclusions can be drawn concerning clinical applications to the population at greatest risk for stroke, the elderly.

## Competing interests

The authors declare that they have no competing interests.

## Authors' contributions

JL drafted the manuscript, LDM organized and edited the manuscript. All authors read and approved the final manuscript.

## References

[B1] Sudlow C, Warlow C (1997). Comparable studies of the incidence of stroke and its pathological types: results from an international collaboration. International Stroke Incidence Collaboration. Stroke.

[B2] Mackay J, Mensch G (2004). The atlas of heart disease and stroke.

[B3] Hurn P, Vannucci S, Hagberg H (2005). Adult or perinatal brain injury: does sex matter?. Stroke.

[B4] McCullough LD, Zeng Z, Blizzard KK, Debchoudhury I, Hurn PD (2005). Ischemic nitric oxide and poly (ADP-ribose) polymerase-1 in cerebral ischemia: male toxicity, female protection. J Cereb Blood Flow Metab.

[B5] McCullough L, Blizzard K, Simpson E, Oz O, Hurn P (2003). Aromatase cytochrome P450 and extragonadal estrogen play a role in ischemic neuroprotection. J Neurosci.

[B6] Anderson G, Limacher M, Assaf A, Bassford T, Beresford S, Black H, Bonds D, Brunner R, Brzyski R, Caan B, Chlebowski R, Curb D, Gass M, Hays J, Heiss G, Hendrix S, Howard BV, Hsia J, Hubbell A, Jackson R, Johnson KC, Judd H, Kotchen JM, Kuller L, LaCroix AZ, Lane D, Langer RD, Lasser N, Lewis CE, Manson J, Margolis K, Ockene J, O'Sullivan MJ, Phillips L, Prentice RL, Ritenbaugh C, Robbins J, Rossouw JE, Sarto G, Stefanick ML, Van Horn L, Wactawski-Wende J, Wallace R, Wassertheil-Smoller S (2004). Effects of conjugated equine estrogen in postmenopausal women with hysterectomy: the Women's Health Initiative randomized controlled trial. JAMA.

[B7] Rosamond W, Flegal K, Friday G, Furie K, Go A, Greenlund K, Haase N, Ho M, Howard V, Kissela B, Kittner S, Lloyd-Jones D, McDermott M, Meigs J, Moy C, Nichol G, O'Donnell CJ, Roger V, Rumsfeld J, Sorlie P, Steinberger J, Thom T, Wasserthiel-Smoller S, Hong Y (2007). Heart disease and stroke statistics–2007 update: a report from the American Heart Association Statistics Committee and Stroke Statistics Subcommittee. Circulation.

[B8] Johnston M, Hagberg H (2007). Sex and the pathogenesis of cerebral palsy. Dev Med Child Neurol.

[B9] Fullerton H, Wu Y, Zhao S, Johnston S (2003). Risk of stroke in children: ethnic and gender disparities. Neurology.

[B10] Marlow N, Wolke D, Bracewell M, Samara M (2005). Neurologic and developmental disability at six years of age after extremely preterm birth. N Engl J Med.

[B11] Nijboer C, Kavelaars A, van Bel F, Heijnen C, Groenendaal F (2007). Gender-dependent pathways of hypoxia-ischemia-induced cell death and neuroprotection in the immature P3 rat. Dev Neurosci.

[B12] Mabley JG, Horvath EM, Murthy KG, Zsengeller Z, Vaslin A, Benko R, Kollai M, Szabo C (2005). Gender differences in the endotoxin-induced inflammatory and vascular responses: potential role of poly(ADP-ribose) polymerase activation. J Pharmacol Exp Ther.

[B13] McCullough L, Hurn P (2003). Estrogen and ischemic neuroprotection: an integrated view. Trends Endocrinol Metab.

[B14] Alonso de Leciñana M, Egido J (2006). Estrogens as neuroprotectants against ischemic stroke. Cerebrovasc Dis.

[B15] Brann D, Dhandapani K, Wakade C, Mahesh V, Khan M (2007). Neurotrophic and neuroprotective actions of estrogen: basic mechanisms and clinical implications. Steroids.

[B16] Cheng J, Alkayed N, Hurn P (2007). Deleterious effects of dihydrotestosterone on cerebral ischemic injury. J Cereb Blood Flow Metab.

[B17] Ryan B, Vandenbergh J (2002). Intrauterine position effects. Neurosci Biobehav Rev.

[B18] Ford G (2008). Clinical pharmacological issues in the development of acute stroke therapies. Br J Pharmacol.

[B19] Savitz S, Fisher M (2007). Future of neuroprotection for acute stroke: in the aftermath of the SAINT trials. Ann Neurol.

[B20] Yamori Y, Horie R, Handa H, Sato M, Fukase M (1976). Pathogenetic similarity of strokes in stroke-prone spontaneously hypertensive rats and humans. Stroke.

[B21] Du L, Bayir H, Lai Y, Zhang X, Kochanek PM, Watkins SC, Graham SH, Clark RS (2004). Innate gender-based proclivity in response to cytotoxicity and programmed cell death pathway. J Biol Chem.

[B22] Renolleau S, Fau S, Goyenvalle C, Joly LM, Chauvier D, Jacotot E, Mariani J, Charriaut-Marlangue C (2007). Specific caspase inhibitor Q-VD-OPh prevents neonatal stroke in P7 rat: a role for gender. J Neurochem.

[B23] Wen T, Rogido M, Peng H, Genetta T, Moore J, Sola A (2006). Gender differences in long-term beneficial effects of erythropoietin given after neonatal stroke in postnatal day-7 rats. Neuroscience.

[B24] Bona E, Hagberg H, Løberg E, Bågenholm R, Thoresen M (1998). Protective effects of moderate hypothermia after neonatal hypoxia-ischemia: short- and long-term outcome. Pediatr Res.

[B25] Pilote L, Dasgupta K, Guru V, Humphries K, McGrath J, Norris C, Rabi D, Tremblay J, Alamian A, Barnett T, Cox J, Ghali WA, Grace S, Hamet P, Ho T, Kirkland S, Lambert M, Libersan D, O'Loughlin J, Paradis G, Petrovich M, Tagalakis V (2007). A comprehensive view of sex-specific issues related to cardiovascular disease. CMAJ.

[B26] Iliescu R, Reckelhoff J (2008). Sex and the kidney. Hypertension.

[B27] Yanes L, Sartori-Valinotti J, Reckelhoff J (2008). Sex steroids and renal disease: lessons from animal studies. Hypertension.

[B28] Cao Z, Liu L, Packwood W, Merkel M, Hurn P, Van Winkle D (2008). Sex differences in the mechanism of Met5-enkephalin-induced cardioprotection: role of PI3K/Akt. Am J Physiol Heart Circ Physiol.

[B29] Bushnell C (2008). Stroke and the female brain. Nat Clin Pract Neurol.

[B30] Fisher M (1999). [The objective of acute stroke therapy and neuroprotective therapeutic approaches]. Rev Neurol.

[B31] (1995). Tissue plasminogen activator for acute ischemic stroke. The National Institute of Neurological Disorders and Stroke rt-PA Stroke Study Group. N Engl J Med.

[B32] Hu BR, Liu CL, Ouyang Y, Blomgren K, Siesjo BK (2000). Involvement of caspase-3 in cell death after hypoxia-ischemia declines during brain maturation. J Cereb Blood Flow Metab.

[B33] Elmore S (2007). Apoptosis: a review of programmed cell death. Toxicol Pathol.

[B34] Rupinder S, Gurpreet A, Manjeet S (2007). Cell suicide and caspases. Vascul Pharmacol.

[B35] Susin S, Daugas E, Ravagnan L, Samejima K, Zamzami N, Loeffler M, Costantini P, Ferri K, Irinopoulou T, Prévost M, Brothers G, Mak TW, Penninger J, Earnshaw WC, Kroemer G (2000). Two distinct pathways leading to nuclear apoptosis. J Exp Med.

[B36] Hoffman G, Merchenthaler I, Zup S (2006). Neuroprotection by ovarian hormones in animal models of neurological disease. Endocrine.

[B37] Heyer A, Hasselblatt M, von Ahsen N, Hafner H, Siren AL, Ehrenreich H (2005). In vitro gender differences in neuronal survival on hypoxia and in 17beta-estradiol-mediated neuroprotection. J Cereb Blood Flow Metab.

[B38] Liu M, Hurn PD, Roselli CE, Alkayed NJ (2007). Role of P450 aromatase in sex-specific astrocytic cell death. J Cereb Blood Flow Metab.

[B39] Renolleau S, Fau S, Charriaut-Marlangue C (2008). Gender-related differences in apoptotic pathways after neonatal cerebral ischemia. Neuroscientist.

[B40] Alkayed N, Harukuni I, Kimes A, London E, Traystman R, Hurn P (1998). Gender-linked brain injury in experimental stroke. Stroke.

[B41] McCullough L, Alkayed N, Traystman R, Williams M, Hurn P (2001). Postischemic estrogen reduces hypoperfusion and secondary ischemia after experimental stroke. Stroke.

[B42] Zhang L, Li PP, Feng X, Barker JL, Smith SV, Rubinow DR (2003). Sex-related differences in neuronal cell survival and signaling in rats. Neurosci Lett.

[B43] Arnold A, Burgoyne P (2004). Are XX and XY brain cells intrinsically different?. Trends Endocrinol Metab.

[B44] Arnold A, Xu J, Grisham W, Chen X, Kim Y, Itoh Y (2004). Minireview: Sex chromosomes and brain sexual differentiation. Endocrinology.

[B45] Carruth L, Reisert I, Arnold A (2002). Sex chromosome genes directly affect brain sexual differentiation. Nat Neurosci.

[B46] Dewing P, Shi T, Horvath S, Vilain E (2003). Sexually dimorphic gene expression in mouse brain precedes gonadal differentiation. Brain Res Mol Brain Res.

[B47] Xu J, Disteche C (2006). Sex differences in brain expression of X- and Y-linked genes. Brain Res.

[B48] Li H, Pin S, Zeng Z, Wang M, Andreasson K, McCullough L (2005). Sex differences in cell death. Ann Neurol.

[B49] Carswell H, Dominiczak A, Macrae I (2000). Estrogen status affects sensitivity to focal cerebral ischemia in stroke-prone spontaneously hypertensive rats. Am J Physiol Heart Circ Physiol.

[B50] Tweel E van den, van Bel F, Kavelaars A, Peeters-Scholte C, Haumann J, Nijboer C, Heijnen C, Groenendaal F (2005). Long-term neuroprotection with 2-iminobiotin, an inhibitor of neuronal and inducible nitric oxide synthase, after cerebral hypoxia-ischemia in neonatal rats. J Cereb Blood Flow Metab.

[B51] Nijboer C, Groenendaal F, Kavelaars A, Hagberg H, van Bel F, Heijnen C (2007). Gender-specific neuroprotection by 2-iminobiotin after hypoxia-ischemia in the neonatal rat via a nitric oxide independent pathway. J Cereb Blood Flow Metab.

[B52] Moroni F (2008). Poly(ADP-ribose)polymerase 1 (PARP-1) and postischemic brain damage. Curr Opin Pharmacol.

[B53] Hagberg H, Wilson MA, Matsushita H, Zhu C, Lange M, Gustavsson M, Poitras MF, Dawson TM, Dawson VL, Northington F, Johnston MV (2004). PARP-1 gene disruption in mice preferentially protects males from perinatal brain injury. J Neurochem.

[B54] Zhu C, Wang X, Huang Z, Qiu L, Xu F, Vahsen N, Nilsson M, Eriksson PS, Hagberg H, Culmsee C, Plesnila N, Kroemer G, Blomgren K (2007). Apoptosis-inducing factor is a major contributor to neuronal loss induced by neonatal cerebral hypoxia-ischemia. Cell Death Differ.

[B55] Culmsee C, Zhu C, Landshamer S, Becattini B, Wagner E, Pellecchia M, Pellechia M, Blomgren K, Plesnila N (2005). Apoptosis-inducing factor triggered by poly(ADP-ribose) polymerase and Bid mediates neuronal cell death after oxygen-glucose deprivation and focal cerebral ischemia. J Neurosci.

[B56] Zhu C, Xu F, Wang X, Shibata M, Uchiyama Y, Blomgren K, Hagberg H (2006). Different apoptotic mechanisms are activated in male and female brains after neonatal hypoxia-ischaemia. J Neurochem.

[B57] Li K, Futrell N, Tovar S, Wang L, Wang D, Schultz L (1996). Gender influences the magnitude of the inflammatory response within embolic cerebral infarcts in young rats. Stroke.

[B58] Fishelson Z, Attali G, Mevorach D (2001). Complement and apoptosis. Mol Immunol.

[B59] Park E, Cho S, Frys K, Glickstein S, Zhou P, Anrather J, Ross M, Iadecola C (2006). Inducible nitric oxide synthase contributes to gender differences in ischemic brain injury. J Cereb Blood Flow Metab.

[B60] Kitano H, Young J, Cheng J, Wang L, Hurn P, Murphy S (2007). Gender-specific response to isoflurane preconditioning in focal cerebral ischemia. J Cereb Blood Flow Metab.

